# Trends and predictions in the physical shape of Chinese preschool children from 2000 to 2020

**DOI:** 10.3389/fpubh.2023.1148415

**Published:** 2023-09-22

**Authors:** Chunjing Tu, Qi Pan, Chongmin Jiang, Yuxuan Tu, Sanhua Zhang

**Affiliations:** ^1^School of Teacher Education (Physical Education), Taizhou University, Taizhou, China; ^2^School of Physical Education, Hangzhou Normal University, Hangzhou, China; ^3^China Institute of Sport Science, Beijing, China; ^4^Department of Polymer Science and Engineering, Zhejiang University, Hangzhou, China; ^5^School of Teacher Education, Hangzhou Normal University, Hangzhou, China

**Keywords:** preschool children, physical shape, GM (1, 1) model, overweight and obesity, longitudinal

## Abstract

**Objective:**

To explore physical shape changes in preschool children from 2000 to 2020, and forecast development trends over the next 10 years.

**Method:**

The grey GM (1,1) prediction model was used to fit the physical shape indicators of preschool children in China from 2000 to 2020, and then the longitudinal change trend of physical shape was compared and analyzed. Finally, the development trend of physical shape in China in 2025 and 2030 was predicted.

**Results:**

(1) During the period from 2000 to 2020, the height, weight and chest circumference of Chinese preschool children all increased rapidly. Specifically, the weight of male and female children increased by 1.8 kg and 1.6 kg, their chest circumference increased by 1.6 cm and 1.5 cm, respectively, and both their heights increased by 3.6 cm. Among these indicators, the older the age, the greater the growth rate. It is expected that all the indicators will continue to grow rapidly over the next 10 years, but the growth rate will slow. (2) From 2000 to 2020, the growth rate of weight was higher than that of height, and BMI showed an increasing trend. The obesity detection rates in boys and girls increased by 5.6 and 2.8%, respectively. Over the next 10 years, the incidence of obesity is expected to increase by 3.8% in boys and 2.8% in girls. (3) Improvement in the growth and development of preschool children in China has a certain correlation with the rapid growth of China’s economy,less physical activity, education and other factors.

**Conclusion:**

Over the past 20 years, the growth and nutritional status of Chinese preschoolers have improved dramatically, but overweight and obesity remain. Overweight and obesity rates are expected to continue to increase rapidly over the next 10 years, particularly among boys, and effective measures should be taken to control the obesity epidemic.

## 1. Introduction

Since China acceded to the World Trade Organization (WTO) in 2001, its economy has grown rapidly, living conditions of its residents have improved dramatically, the level of nutrition has improved, and the growth and development of children has increased (1). However, the rates of overweight and obesity have increased significantly (2, 3). Obesity is a serious public health problem threatening children in many countries and regions (4). Research shows that from 1975 to 2016, the average BMI of boys and girls aged 5–19 increased by 0.40 kg/m^2^ and 0.32 kg/m^2^ per decade in worldwide (5), respectively. In a study of American children and adolescents,Odgen pointed out that the obesity rate of children aged 2–5 years increased from 7.2% in the 1988–1994 period to 13.9% in the 2003–2004 period, then dropped to 12.1% in the 2009–2010 periodand 9.4% in the 2013–2014 period, showing a downward trend (6). Globally, the growth rate of obesity in developed countries is decreasing, whereas the number of obese children and adolescents in developing countries continues to increase (7). China is a typical developing country in which preschool children are facing a severe obesity epidemic (8).

Preschool is a fundamental stage in the development of good physical fitness, and the level of growth and development during this period has an important impact on adolescence, adulthood, and old age (9–11). Exploring changes and trends in growth and development can provide references for preschool children to improve their physical fitness and health and prevent chronic diseases. At present, Chinese scholars’ research on the growth and development of preschool children mainly focuses on cross-sections, and longitudinal research only focuses on individual cities; most longitudinal research methods are simple comparisons or linear regression analyses of different ages (12, 13). However, there has been no in-depth nationwide research on the longitudinal dynamic changes and predictions of body shape and obesity. The grey model GM (1, 1) is an effective model for fuzzy long-term descriptions of the development of things (14, 15). Since its establishment in 1982, the gray model has been widely used in many fields such as medicine (16), economy (17), agriculture (18), and sports (19); successfully solving a large number of practical problems in production, life, and scientific research.

China attaches great importance to the physical health of children. Since 2000, China has implemented a national physical fitness surveillance program, and established a five-year cycle of institutional and systematic national physical fitness surveillance systems. The country has conducted five national physical fitness surveys (in 2000, 2005, 2010, 2014, and 2020) covering 31 provinces (autonomous regions and municipalities). This national physical fitness surveillance includes physical shape indicators such as height, weight, chest circumference, and BMI of children aged 3–6 years. These surveys provide important data for studying children’s growth and development in China.

Based on the body shape data of preschool children obtained from five national fitness surveys, this study established a grey prediction model GM (1, 1) for body shape series indicators to explore the internal laws and trends of the growth and development of Chinese school-age children. and to provide the scientific basis for government departments, kindergartens, and families to formulate targeted intervention measures to improve the growth and development level of preschool children and curb the epidemic of overweight and obesity in China.

## 2. Materials and methods

### 2.1. Research subjects

The participants were preschool children aged 3–6 years in China. The data was derived from five China national physical fitness surveillances (CNPFS) (20–24). Each survey selected a nationally representative sample of civilians. The sampling scheme is as follows: the first stage covered all 31 provinces, autonomous regions, and municipalities in mainland China. In the second stage, three cities were randomly selected from each province according to their economic condition assessed by GDP; in the third stage, three districts (for urban area) and three counties (for rural area) from each city; in the fourth stage, three streets/towns from each district/county; in the fifth stage, two kindergarten from each street/town. The last stage adopts the method of systematic sampling; they were divided into eight groups according to gender and age, and each group spot tests 100 (2000 is 80 people) in each province. Assuming a response rate of 100/130 with expected enrollment of 1,600 (2020 is 1,280 people) for each province, a total of 52,687, 53,966, 50,546, and 49,667 boys and girls between age 3 ~ 6 were surveyed in 2000, 2005, 2010, 2014, and 2020, respectively; and total of 247,088 valid samples were obtained in altogether.

### 2.2. Measurement method

All tests were conducted at the same location with the same brand of testing equipment (Jianmin II, Beijing)and the same testing methods. Height was measured to the nearest 0.1 cm using a calibrated height measurement instrument. Weight was measured with a corrected weight measurement instrument, accurate to 0.1 kg. Chest circumference measurements were taken horizontally, and the lower margin of the nylon tape ruler on the chest along the upper margin of the nipple was accurate to 0.1 cm. BMI = weight/(height × height) (kg/m^2^). Parents or guardians were given a sports medicine questionnaire before they were tested, on the promise that parents had learned, consented, and signed the consent forms. The tester checked the subjects who participated in this experiment actively, and excluded children with diseases of the heart, lungs, liver, kidneys, and other major organs (such as a history of heart disease, asthma, hyperthyroidism, or other physical disabilities) or other children who were not suitable to participate in more strenuous sports.

### 2.3. Research method

The main research method of this study uses the data obtained from previous monitoring as time-series data, constructs the grey GM (1, 1) model, develops the fitted curves of growth and development indicators, analyzes the dynamic changes in growth and development during the 20 years from 2000 to 2020, and finally predicts the values of each indicator in 2025 and 2030,The grey GM (1, 1) model is described as follows:

#### 2.3.1. Introduction to the grey model GM (1,1)

Grey systems theory, established by Professor Julong Deng in 1982, is a relatively newmethodology that focuses on the study of problems involving small data and poorinformation (14), Grey system theory is one of the three new uncertainty theories (fuzzy mathematics, grey system, and rough set) in the second half of the 21st century (15). The two SCI regional journals “Grey Systems Theory and Application” and “Journal of Grey System” established by the Institute of Grey Systems provide an important platform for the exchange and development of this theory in the past 40 years, grey system theory has been developed rapidly (25). A large number of scholars, such as LotfiA Zadeh, founder of fuzzy mathematics, Robert Valee, president of the World Organization for Systems and Control, have highly praised the research on grey systems. German Chancellor Angela Merkel praised China’s original grey system theory in a speech at Huazhong University of Science and Technology in 2019, believing that the theory and related work has “profoundly affected the world” (25, 26).

GM (1, 1) model is a prediction model of grey system theory and an important branch of grey system theory with the most classic results (27). GM (1, 1) represents a Grey Model of a first-order differential equation with one variable, where G represents grey and M represents Model, the first 1 in parentheses represents the first-order differential equation, and the second 1 represents the equation with only one independent variable, GM (1, 1) has the characteristics of “less series data,” “isochronous” and “time series,” and the time series of no less than 4 data can build the prediction model (28). The modeling principle of GM (1, 1) is to first process the existing gray information extraction, processing, generation and other information, mining the internal change law of the system, and then make a quantitative prediction of the future trend. The model parameter development coefficient a ≥ − 0.3 can be used for short and medium term prediction, and has a high accuracy (15).

The method and results of this study are reliable and repeatable. A computer software on GM (1, 1) can be downloaded from the web site of Institute for Grey System Studies of Nanjing University of Aeronautics and Astronautics^1^ for free (29).

#### 2.3.2. Model building process

The main process of the grey GM (1, 1) model construction is to generate equidistant time-series data, establish a prediction model, test the model, and obtain the fitted and predicted values (15). The height of a 3-year-old male child was used as an example to illustrate the modeling prcess.

The five national monitoring heights in 2000, 2005, 2010, 2014, and 2020 were 99.1 cm, 100.2 cm, 101.3 cm, 102.1 cm and 101.9 cm, respectively. According to the same time intervals modeling requirements, 102.1 cm in 2014 was adjusted to 102.3 cm in 2015 by interpolation. Since the first data of the modeled series does not affect the predicted values and development coefficients of the model (16), an arbitrary value “10” is added before this time series in order to make full use of the original data and to improve the accuracy and reliability of the predictions. Therefore, the initial vector of modeling is (10,99.1, 100.2, 101.3, 102.3, 101.9). The specific steps are described as follows:

1. The initial vector is: $\;X^{\left(0\right)}\left(m\right)=\left(10,\;\;99.1,\;\;100.2,\;\;101.3,\;\;102.3,\;\;101.9\right)$

2. Accumulating Generation Operator (1-AGO) for the initial vector:

$$X^{\left(1\right)}\left(m\right)=\left[10,\;\;109.1,\;\;209.3,\;\;310.6,\;\;\;412.9,\;\;\;514.8\right]$$

3. 1-AGO generates the adjacent mean of the sequence

$$W^{\left(0\right)}\left(m\right)=\left[59.55,\;\;159.20,\;\;259.95,\;\;361.75,\;\;463.85\right]$$

4. Generate the coefficient matrix *B* and *Y* (n):

$B=\left[\begin{array}{c} \begin{array}{cc} -59.55 & 1\\ -159.20 & 1\\ -259.95 & 1 \end{array}\\ \begin{array}{cc} -361.75 & 1\\ -463.85 & 1 \end{array} \end{array}\right]$ , Y(n)=[99.1,100.2,101.3,102.3,101.9].

5. Least squares method to solve for the grey model development coefficients a and constant b:

$$\left(\begin{array}{c} a\\ b \end{array}\right)={\left(B^{T}B\right)}^{-1}\;BY^{T}=\left(\begin{array}{c} -0.00760\\ 98.97662 \end{array}\right)$$

6. After substituting a and b into the response function, derivation is obtained and the prediction model is restored:

$$Y^{\left(0\right)}\left(m\right)=\left(1-e^{a}\right)\left(X^{\left(0\right)}\left(1\right)-\frac{b}{a}\right)e^{-a\left(m-1\right)}=98.6772e^{0.0076\left(m-1\right)}$$

7. The calculated average simulation relative error is 0.36764%, the average relative precision is 99.63236% (See Table 1), and the mean square error ratio C is 0.37 (see Table 2); This shows that the effectiveness is good (Level 2).

**Table 1:** Statistical list of the relative error between predicted simulated and original value in this study.

Annual	Original value	Fit values, Predicted values	Residual	Precision of fitting
2000	99.1	99.4	−0.3	99.7%
2005	100.2	100.2	0.1	99.9%
2010	101.3	101.0	0.3	99.7%
2015	102.3	101.7	0.6	99.4%
2020	101.9	102.5	−0.6	99.4%
2025	/	103.3	/	/
2030	/	103.8	/	/

**Table 2:** A list of testing standards for the grey forecasting model in this study.

Model fit rank	Ratio C of mean squared variance	Small error probability P	Average relative accuracy
Excellent (level 1)	<3.5e-01	>9.5e-01	>99.0%
Good (level 2)	<5.0e-01	>8.0e-01	>95.0%
Qualified (Level 3)	<6.5e-01	>7.0e-01	>90.0%
Nonconforming standard (Level 4)	<8.0e-01	>6.0e-01	≤90.0%

## 3. Research result

### 3.1. Sample characteristics

The main physical shape indicators were height, weight, and chest circumference. The results showed that the growth and development levels of all the age groups increased with age. From age 3 to 6, the greatest increase was observed in weight. All indicators were higher in boys than in girls across all age groups (see Table 3).

**Table 3:** Characteristic values of samples of children aged 3 to 6 in China from 2000 to 2020.

Annual	Indicators	Boys				Girls			
		3 years old	4 years old	5 years old	6 years old	3 years old	4 years old	5 years old	6 years old
	Height	99.1 ± 5.4	105.2 ± 5.5	111.0 ± 5.9	115.6 ± 5.9	98.0 ± 5.4	104.0 ± 5.5	109.9 ± 5.8	114.4 ± 5.8
2000	Weight	15.5 ± 2.0	17.2 ± 2.4	19.0 ± 3.0	20.6 ± 3.3	14.9 ± 2.0	16.5 ± 2.3	18.2 ± 2.7	19.6 ± 3.0
	C-C	51.7 ± 2.9	53.2 ± 3.0	54.8 ± 3.4	56.0 ± 3.6	50.5 ± 3.1	51.8 ± 3.1	53.2 ± 3.3	54.4 ± 3.6
	BMI	15.8 ± 1.5	15.5 ± 1.4	15.4 ± 1.6	15.3 ± 1.6	15.5 ± 1.5	15.2 ± 1.5	15.0 ± 1.4	14.9 ± 1.5
	Height	100.2 ± 5.3	106.3 ± 5.2	112.4 ± 5.9	117.5 ± 5.6	99.1 ± 5.3	105.1 ± 5.2	111.0 ± 5.7	116.1 ± 5.5
2005	Weight	16.0 ± 2.3	17.7 ± 2.7	19.7 ± 3.4	21.6 ± 3.9	15.4 ± 2.1	16.9 ± 2.4	18.8 ± 3.0	20.5 ± 3.5
	C-C	51.8 ± 2.9	53.2 ± 3.2	55.1 ± 3.6	56.6 ± 4.2	50.7 ± 3.0	51.8 ± 3.1	53.3 ± 3.7	54.7 ± 4.0
	BMI	15.9 ± 1.6	15.6 ± 1.6	15.5 ± 1.7	15.5 ± 1.5	15.6 ± 1.5	15.3 ± 1.5	15.2 ± 1.7	15.1 ± 1.8
	Height	101.3 ± 5.0	107.1 ± 5.3	113.7 ± 5.5	118.5 ± 5.7	99.9 ± 5.2	105.9 ± 5.1	112.4 ± 5.4	117.0 ± 5.6
2010	Weight	16.4 ± 2.4	18.1 ± 2.9	20.5 ± 3.6	22.5 ± 4.3	15.7 ± 2.2	17.4 ± 2.7	19.5 ± 3.2	21.1 ± 3.6
	C-C	52.5 ± 3.0	54.0 ± 3.4	56.0 ± 3.9	57.5 ± 4.4	51.3 ± 3.0	52.6 ± 3.3	54.2 ± 3.6	55.5 ± 4.1
	BMI	16.0 ± 1.7	15.7 ± 1.7	15.8 ± 1.8	15.9 ± 2.1	15.7 ± 1.6	15.5 ± 1.7	15.3 ± 1.7	15.3 ± 1.8
	Height	102.1 ± 5.4	107.8 ± 5.2	114.0 ± 5.4	119.7 ± 5.5	100.8 ± 5.2	106.5 ± 5.2	112.7 ± 5.3	118.1 ± 5.5
2014	Weight	16.6 ± 2.4	18.3 ± 2.8	20.6 ± 3.5	23.0 ± 4.4	15.9 ± 2.1	17.5 ± 2.6	19.6 ± 3.2	21.6 ± 3.6
	C-C	52.9 ± 3.1	54.5 ± 3.4	56.3 ± 3.9	58.2 ± 4.5	51.8 ± 3.0	53.1 ± 3.2	54.6 ± 3.7	56.2 ± 3.9
	BMI	15.8 ± 1.6	15.7 ± 1.6	15.8 ± 1.9	16.0 ± 2.1	15.6 ± 1.5	15.4 ± 1.6	15.4 ± 1.8	15.4 ± 1.8
	Height	101.9 ± 5.2	108.0 ± 5.0	115.3 ± 5.3	119.6 ± 5.4	100.9 ± 5.2	107.0 ± 5.1	114.1 ± 5.2	118.5 ± 5.4
2020	Weight	16.4 ± 2.3	18.4 ± 2.5	21.4 ± 2.9	23.1 ± 3.2	15.8 ± 2.4	17.7 ± 2.5	20.2 ± 2.8	21.9 ± 3.1
	C-C	52.3 ± 3.1	54.1 ± 3.5	56.3 ± 3.7	58.1 ± 4.1	51.2 ± 3.2	52.7 ± 3.5	54.6 ± 3.7	56.1 ± 4.0
	BMI	15.8 ± 1.6	15.8 ± 1.8	16.1 ± 1.7	16.2 ± 1.9	15.8 ± 1.5	15.5 ± 1.7	15.5 ± 1.7	15.6 ± 1.8

### 3.2. Dynamic changes and prediction of body morphology from 2000 to 2020

Using the Grey model GM (1, 1) to predict the body shape indicators of 3-, 4-, 5-, and 6-year-old children, the parameters, fitting values, and predicted values of the prediction model were obtained. The value of the development coefficient of the model parameter “a” reflects the intrinsic development trend of the time series data; if “a” is “negative,” it means the increasing trend of the series, if “a” is “positive,” it indicates a decreasing trend. The size of the absolute value of “a” indicates the magnitude of increase or decrease; and the smaller the absolute value, the smaller the magnitude of change in the time series, and vice versa (15, 30).

#### 3.2.1. Height

According to the relevant parameters of the height grey model GM (1, 1) (see Table 4), the average error of the fitting curve for each age is 0.14% ~ 0.43%, and the accuracy of the models is above 99.5%, indicating that the established models are highly effective. The development coefficient a of all age groups was negative, and the values of |a| were 5 years >6 years >3 years >4 years. (1) From 2000 to 2020, the height of boys aged 3, 4, 5, and 6 increased by 3.1 cm, 2.9 cm, 4.1 cm and 4.1 cm, respectively; with an average increase of 3.6 cm (an annual increase of 0.16%). The height of girls increased by 3.1 cm, 3.0 cm, 4.1 cm and 4.2 cm, respectively; with an average increase of 3.6 cm (an annual increase of 0.17%). It can be seen that the growth rate of 5- and 6-year-olds is higher than that of 3- and 4-year-olds, with no gender difference. (2) It is predicted that during 2020 to 2030, the height of boys aged 3, 4, 5, and 6 will increase by 1.3 cm, 1.2 cm, 1.9 cm and 1.7 cm, respectively, with an average increase of 1.5 cm (an annual increase of 0.14%). The height of girls will increase by 1.4 cm, 1.4 cm, 2.0 cm and 1.8 cm, respectively, with an average increase of 1.7 cm (an annual increase of 0.15%). As a result, the height of Chinese preschool children will continue to increase over the next 10 years, but the annual growth rate will be slightly lower than that of the previous 10 years.

**Table 4:** Parameters, fitting and predicted values of GM (1, 1) for height of preschool children in China, 2000–2030.

Parameter and annual	Boys				Girls			
	3 years old	4 years old	5 years old	6 years old	3 years old	4 years old	5 years old	6 years old
10-3a	−7.6	−6.8	−9.1	−8.8	−7.7	−7.2	−9.1	−8.9
b	99.0	105.0	110.7	115.6	97.8	103.8	109.5	114.1
Fitting error	0.4%	0.3%	0.2%	0.4%	0.3%	0.2%	0.1%	0.3%
2000	99.4	105.5	111.3	116.2	98.2	104.2	110.0	114.8
2005	100.2	106.2	112.3	117.2	99.0	105.0	111.0	115.8
2010	101.0	106.9	113.3	118.2	99.8	105.7	112.0	116.9
2015	101.7	107.6	114.3	119.3	100.6	106.5	113.0	117.9
2020	102.5	108.4	115.4	120.3	101.3	107.2	114.1	119.0
2025	103.3	109.1	116.4	121.4	102.1	108.0	115.1	120.0
2030	103.8	109.6	117.3	122.0	102.7	108.6	116.1	120.8
2020–2000	3.1(3.1%)	2.9(2.7%)	4.1(3.7%)	4.1(3.5%)	3.1(3.2%)	3.0(2.9%)	4.1(3.7)	4.2(0.3.7%)
2030–2020	1.3(1.3%)	1.2(1.1%)	1.9(1.6%)	1.7(1.4%)	1.4(1.4%)	1.4(1.3%)	2.0(1.8%)	1.8(1.5%)

#### 3.2.2. Weight

According to the relevant parameters of the weight gray model GM (1, 1) (see Table 5), the average error of the fitting curve of each age is 0.5% ~ 1.3%, and the accuracy of the models is above 98.5%, indicating that the established models are highly effective. The development coefficient a of all age groups was negative, and the values of |a| were 6 years >5 years >4 years >3 years. (1) From 2000 to 2020, the weight of boys aged 3, 4, 5, and 6 increased by 1.0 kg, 1.3 kg, 2.3 kg, and 2.6 kg, respectively; with an average increase of 1.8 kg (an annual increase of 0.46%). The weight of girls increased by 0.9 kg, 1.2 kg, 1.9 kg, and 2.3 kg, respectively; with an average increase of 1.6 kg (an annual increase of 0.4%). The growth rate of boys was higher than that of girls; and the older the age, the faster the rate of weight gain. (2) It is predicted that during 2020–2030, the weight of boys aged 3, 4, 5 and 6 will increase by 0.3 kg, 0.5 kg, 1.1 kg, and 1.2 kg, respectively, with an average increase of 0.8 kg (an annual increase of 0.4%). The weight of girls will increase by 0.3 kg, 0.6 kg, 1.0 kg, and 1.1 kg, respectively, with an average increase of 0.7 kg (an annual increase of 0.4%). As a result, the weight of Chinese preschool children will continue to increase over the next 10 years, but the annual growth rate will be slightly lower than that of the previous decade.

**Table 5:** Parameters, fitting and predicted values of GM (1, 1) for weight of preschool children in China, 2000–2030.

Parameter and annual	Boys				Girls			
	3 years old	4 years old	5 years old	6 years old	3 years old	4 years old	5 years old	6 years old
10-3a	−14.7	−17.2	−28.1	−29.4	−14.7	−17.4	−24.8	−27.6
b	15.4	17.0	18.6	20.3	14.8	16.3	14.8	19.1
Fitting error	1.2%	0.7%	0.6%	1.3%	1.0%	0.5%	0.6%	0.8%
2000	15.7	17.3	19.1	20.9	15.1	16.6	18.3	19.8
2005	15.9	17.6	19.7	21.5	15.3	16.9	18.8	20.4
2010	16.2	18.0	20.2	22.2	15.5	17.2	19.2	20.9
2015	16.4	18.3	20.8	22.8	15.8	17.5	19.7	22.5
2020	16.7	18.6	21.4	23.5	16.0	17.8	20.2	22.1
2025	16.9	18.9	22.0	24.2	16.2	18.1	20.7	22.7
2030	17.0	19.1	22.5	24.7	16.3	18.4	21.2	23.2
2020–2000	1.0(6.4%)	1.3(7.5%)	2.3(12.0%)	2.6(12.4%)	0.9(6.0%)	1.2(7.2%)	1.9(10.4%)	2.3(11.6%)
2030–2020	0.3(1.8%)	0.5(2.7%)	1.1(5.1%)	1.2(5.1%)	0.3(1.9%)	0.6(3.4%)	1.0(5.0%)	1.1(5.0%)

#### 3.2.3. Chest circumference

According to the relevant parameters of the chest circumference gray model GM (1, 1) (see Table 6), the average error of the fitting curve of each age is 0.4% ~ 0.8%, and the accuracy of the models is above 99.0%, indicating that the established models are highly effective. The development coefficient a of all age groups is negative, and the value of |a| is 6 years >5 years >4 years >3 years. (1) From 2000 to 2020, the chest circumference of boys aged 3, 4, 5, and 6 increased by 0.9 cm, 1.3 cm, 1.7 cm, and 2.4 cm, respectively; with an average increase of 1.6 cm (an annual increase of 0.14%). The chest circumference of girls increased by 1.1 cm, 1.3 cm, 1.7 cm, and 2.0 cm respectively; with an average increase of 1.5 cm (an annual increase of 0.1%). It can be seen that the growth rate of 5- and 6-year-olds is higher than that of 3- and 4-year-olds. (2) It is predicted that during 2020–2030, the chest circumference of boys aged 3, 4, 5, and 6 will increase by 0.5 cm, 0.6 cm, 0.8 cm, and 1.2 cm, respectively, with an average increase of 0.8 cm (an annual increase of 0.1%). The chest circumference of girls will increase by 0.5 cm, 0.6 cm, 0.9 cm, and 1.0 cm, respectively, with an average increase of 0.8 cm (an annual increase of 0.1%). As a result, the chest circumference of Chinese preschool children will continue to increase over the next 10 years, with the same annual growth rate as in the last decade.

**Table 6:** Parameters, fitting and predicted values of GM (1, 1) for chest circumference of preschool children in China, 2000–2030.

Parameter and annual	Boys				Girls			
	3 years old	4 years old	5 years old	6 years old	3 years old	4 years old	5 years old	6 years old
10-3a	−4.6	−5.9	−7.7	−10.5	−5.3	−6.1	−8.0	−9.0
b	51.6	53.0	54.6	55.7	50.4	51.6	52.9	54.1
Fitting error	0.8%	0.5%	0.4%	0.5%	0.6%	0.5%	0.4%	0.4%
2000	51.8	53.2	54.9	56.1	50.6	51.8	53.2	54.4
2005	52.0	53.5	55.3	56.7	50.9	52.1	53.6	54.9
2010	52.3	53.8	55.7	57.3	51.1	52.4	54.0	55.4
2015	52.5	54.1	56.1	57.9	51.4	52.7	54.4	55.9
2020	52.7	54.5	56.6	58.5	51.7	53.1	54.9	56.4
2025	53.0	54.8	57.0	59.1	52.0	53.4	55.3	56.9
2030	53.2	55.1	57.4	59.7	52.2	53.7	55.8	57.4
2020–2000	0.9(1.7%)	1.3(2.4%)	1.7(3.1%)	2.4(4.3%)	1.1(2.2)	1.3(2.5)	1.7(3.2)	2.0(3.7)
2030–2020	0.5(1.0%)	0.6(1.1%)	0.8(1.4%)	1.2(2.1%)	0.5(1.0%)	0.6(1.1%)	0.9(1.6)	1.0(1.8%)

#### 3.2.4. BMI

According to the relevant parameters of the BMI gray model GM (1, 1) (see Table 7), the average error of the fitting curve of each age is 0.2% ~ 0.4%, and the accuracy of the models is above 99.5%, indicating that the established models are highly effective. The development coefficient a of all age groups is negative, and the value of |a| is 6 years >5 years >4 years >3 years. (1) From 2000 to 2020, the BMI of boys aged 3, 4, 5, and 6 increased by −0.1, 0.3, 0.6, and 0.9%, respectively, with an average increase of 0.4 (annual increase of 0.14%), while that of girls increased by 0.2, 0.3, 0.4, and 0.7%, respectively, with an average increase of 0.4 (annual increase of 0.13%). It can be seen that the growth rate of 6- and 5-year-olds were higher than that of 4- and 3-year-olds. (2) Between 2020 and 2030, the BMI of boys aged 3, 4, 5, and 6 years are predicted to increase by 0, 0.2, 0.4, and 0.5, respectively, with an average increase of 0.3 (an annual increase of 0.17%). The BMI of girls will increase by 0.1, 0.1, 0.2, and 0.4, respectively, with an average increase of 0.2 (an annual increase of 0.13%). As a result, the BMI of Chinese preschool children will continue to increase in the next 10 years, and the annual growth rate will be higher for boys than in the previous decade.

**Table 7:** Parameters, fitting and predicted values of GM (1, 1) for BMI of preschool children in China, 2000–2030.

Parameter and annual	Boys				Girls			
	3 years old	4 years old	5 years old	6 years old	3 years old	4 years old	5 years old	6 years old
10-3a	−0.4	−4.7	−10.6	−14.2	−3.4	−4.4	−7.5	−11.2
b	15.8	15.4	15.2	15.0	15.5	15.2	14.9	14.7
Fitting error	0.4%	0.2%	0.4%	0.2%	0.4%	0.3%	0.3%	0.2%
2000	15.9	15.5	15.4	15.3	15.5	15.2	15.1	14.9
2005	15.9	15.6	15.5	15.6	15.6	15.3	15.2	15.1
2010	15.9	15.7	15.7	15.8	15.6	15.4	15.3	15.2
2015	15.8	15.7	15.9	16.0	15.7	15.4	15.4	15.5
2020	15.8	15.8	16.0	16.2	15.7	15.5	15.5	15.6
2025	15.8	15.9	16.2	16.5	15.8	15.6	15.6	15.8
2030	15.8	16.0	16.4	16.7	15.8	15.6	15.7	16.0
2020–2000	−0.1(−0.6%)	0.3(1.9%)	0.6(3.9%)	0.9(5.9%)	0.2(1.3%)	0.3(2.0%)	0.4(2.7%)	0.7(4.7%)
2030–2020	0.0(0.0%)	0.2(1.3%)	0.4(2.5%)	0.5(3.1%)	0.1(0.6%)	0.1(0.7%)	0.2(1.3%)	0.4(2.6%)

## 4. Discussion

### 4.1. Preschool children body shape change characteristics and trend prediction

In this research, we found that the level of growth of Chinese Preschool Children improved evidently from 2000 to 2020; each indication continued to grow rapidly, and the average annual increase was 0.12% ~ 0.44% in height, weight, and chest circumference. Comparison of the average increase is as follows: weight > height > chest circumference. After stratification by age, it was found that the older the age, the greater the growth rate. In terms of development trends, both male and female children’s body shape indicators will continue to keep growing from 2020 to 2030; but the annual increase will be slightly lower than that from 2000 to 2020. For example, for 6-year-old boys, the height increased by 2.0 cm, 2.1 cm, and 1.5 cm per decade from 2000 to 2010, 2010–2020, and 2020–2030, respectively; and the weight increased by 1.3 kg, 1.3 kg, and 1.2 kg. Tracing the relevant studies before 2000, starting from 1975, the Chinese Ministry of Health organized a physical survey of children aged 0–7 years in nine major cities every 10 years, ^[13]^its conclusion shows that the height of 6-year-old children increased by 1.7 cm and 2.1 cm, and their weight increased by 0.4 kg and 1.1 kg per decade in the 1975–1985 and 1985–1995 periods, respectively. It can be seen that during 1975–2020, height and weight went from medium growth (1975–1985) to high growth (1985–2020) and will continue to grow at a medium rate in the next 10 years (2020–2030).

### 4.2. Analysis of causes of changes in growth and development

Since its reform and opening up in 1978, China’s economy has undergone rapid development, with its *per capita* GDP growing from 385 yuan in 1978 to 71,828 yuan by 2020. From 2000 to 2020, China’s per-capita GDP increased from 7,942 yuan to 71,828 yuan (an increase of 8.0 times), the disposable income of residents increased from 3,721 yuan to 32,189 yuan (an increase of 7.7 times), and the Engel coefficient decreased from 42.2 to 29.3%. Economic development has resulted in increased living standards and changes in dietary structure (more abundant foods that promote physical growth and development, such as meat, eggs, and milk). The correlation analysis between the growth values of height and weight and national economic data (31) from 2000 to 2020 shows that the correlation coefficients of height with GDP *per capita* (12, 32), disposable income *per capita* and Engel index are 0.22, 0.23 and − 0.46, respectively; and the correlation coefficients of weight with the same are 0.45, 0.42 and − 0.38, respectively. Therefore, growth and development are correlated with socioeconomic and consumption structures, and domestic studies have shown that body shape is positively correlated with GDP. Since the 1950s, there has been a significant increase in the height, weight, and chest circumference of children in most developed countries, such as Korea and Japan. Korea’s GDP *per capita* increased from $87 in 1962 to $10,548 in 1996, and the height of children increased by 5 cm between 1965 and 2005 (an average annual increase of 0.13 cm); further segmentation shows that Korean children’s height increased rapidly from 1965 to 1996, while it maintained a low growth rate from 1997 to 2005 (33). The Japanese economy went through periods of high growth (1955–1973), moderate growth (1974–1991), and stagnation (after 1992); and child growth and development also went through periods of high, moderate, and low stability. For example, the height of 6-year-old children in Japan increased by 2.2 cm, 1.5 cm, and 0.6 cm per decade from 1963 to 1993, respectively; but most of the increase occurred before 1980, while there was little change from 1993 to 2010 (12, 34). suggesting that the long-term trend in height has stopped. It can be seen that in the past few decades, Japan, Korea, and China have experienced rapid economic development, which corresponds to a significant increase in child growth and development, showing that the changes in growth and development levels are related to the factors of economic development levels, but also that the impact of socio-economic changes on physical morphological development shows stages; when growth and development reach a certain level, its growth rate is bound to slow down or even stagnate. Moreover, records show that the mean height in the Netherlands has increased since 1858; however, this growth stopped after 1997 (35). Our study predicts a slowdown in height growth in China over the next 10 years, similar to the slowdown after high growth in countries such as Japan, Korea, and the Netherlands. Therefore, when economic development is at a higher level, its impact on children’s growth and development gradually decreases.

### 4.3. Prevalence of obesity among preschool children in China

This study found that from 2000 to 2020, preschool children gained more weight than height, with BMI increasing by 0.4 kg/m^2^ (0.14% annual increase) and 0.4 kg/m^2^ (0.13% annual increase), respectively, and body fullness increasing. For the forecast period 2020–2030, BMI is predicted to increase by 0.43 kg/m^2^ (0.17% *per annum*) and 0.2 kg/m^2^ (0.13% *per annum*) for boys and girls, respectively.

Increased growth and development has been accompanied by an obesity epidemic. To further analyze the changes and trends in the body shape of preschool children over the past 20 years, a study was conducted on the detection rates of overweight and obesity over time. First, by referring to the standards of overweight and obesity thresholds for young children aged 3–6 years in China (19), the author counted the detection rates of overweight and obesity for children in this age group in China in 2000, 2005, 2010 and 2014 (see Table 8). The results showed that the overweight detection rates over the years for male children were 9.6, 10.3, 12.0, and 12.1%, and the obesity rates were 5.4, 7.5, 8.8, and 9.5%, respectively. Overweight detection rates over the years for girls were 7.0, 8.2, 9.1, and 9.6%, and obesity rates were 3.4, 4.7, 5.2, and 5.7%, respectively. Second, based on the above historical monitoring data, predictions using the GM (1, 1) model show that the overweight and obesity rates in 2030 will be 14.7 and 14.1% for male children, and 12.8 and 7.4% for female children, respectively. Finally, between 2000 and 2020, the rates of overweight and obesity increased by 3.5 and 5.6% for male children, and 3.8 and 2.8% for female children, respectively. From 2020 to 2030, the overweight and obesity rates are projected to increase by 1.6 and 3.1% for males and 1.9 and 1.2% for females, respectively. The rate of overweight and obesity among preschool children will continue to increase at a faster rate, with preschool children facing an increasingly serious obesity situation. The results of a survey in nine large Chinese cities (the study referred to WHO criteria for overweight and obesity) (36) showed that an epidemic of childhood obesity had not yet developed in China in the 1980s, but there has been a growing trend since the 1990s. In 1996, the obesity detection rates for boys and girls aged 0–7 years were 2.12 and 1.38%, respectively. In 2006, the obesity detection rates for boys and girls aged 0–7 years were 3.82 and 2.48%, respectively. This study suggests that overweight and obesity rates among preschool children will continue the increasing trend, observed since the 1990s, in the 2020–2030 period.

**Table 8:** Overweight and obesity detection rate of 3 ~ 6-year-old children in China, 2000–2030 (%).

Project	2000	2005	2010	2014	2020	2025	2030	2020–2000	2030–2020
Overweight rate of boys	9.6	10.3	12.0	12.1	13.1	14.0	14.7	3.5	1.6
Obesity rate of boys	5.4	7.5	8.8	9.5	11.0	12.5	14.1	5.6	3.1
Overweight rate of girls	7.0	8.2	9.1	9.6	10.9	11.8	12.8	3.8	1.9
Obesity rate of girls	3.4	4.7	5.2	5.7	6.2	6.7	7.4	2.8	1.2

The obesity epidemic among preschool children is caused by several factors maybe. First, it is related to the growth of the economy; first, the improvement of life conditions leads to consuming large amounts of high-calorie foods; second, there is less time for physical activity and preschool children lead a lifestyle of less activity, with more time spent on TV, video games and computers, and participating in training courses for mathematics, foreign languages, and painting. An imbalance in energy intake and consumption caused by these changes may be an important cause of obesity. Preschool children have a relatively limited autonomous choice of diet and lifestyle owing to their young age, and external factors such as family income, parental education, and educational environment play a more significant role (37). Preschool is critical for preventing and healing obesity (38). Thus, parents and kindergartens should actively contribute to curbing the obesity epidemic in preschool children.

## 5. Conclusion

Our data from five waves of large-scale surveys (CNPFS) over a 20-year period, which included 247,088 nationally representative sample, has a large sample size with strong representativeness. The growth and development level of preschool children greatly improved during the period from 2000 to 2020, and will continue to maintain relatively fast growth in the next 10 years, but the growth rate is slightly lower than that of the previous 20 years. At the same time, if the increase in weight is greater than that in height,BMI will continue to increase in the next 10 years, and the detection rate of overweight and obesity will continue to increase. So the government should scientifically study and judge the development direction of the work related to the growth and development of preschool children, and rationally plan and deploy the work priorities,and Families and kindergartens should focus on the nutritional balance of preschool children and guide them to increase physical activity and reduce resting behavior, to development level of preschool children and curb the epidemic of overweight and obesity in China.

## Data availability statement

The original contributions presented in the study are included in the article/supplementary material, further inquiries can be directed to the corresponding author.

## Ethics statement

Ethical issues (including plagiarism, informed consent, misconduct, data fabrication and/or falsification, double publication and/or submission, and redundancy) were observed by the authors.

## Author contributions

CT did the overall trial management. CJ conceived and designed the study. QP, YT, and SZ contributed to the study analysis plan and data analysis. All authors contributed to the article and approved the submitted version.

## Funding

This study was supported by the Key Project of the Ministry of Education of the People’s Republic of China in the 13th Five-Year Plan for Education under Grant No. DHA180359, and Zhejiang Provincial Philosophy and Social Science Planning Project under Grant No.19NDJC061YB and No.19NDJC059YB.

## Conflict of interest

The authors declare that the research was conducted in the absence of any commercial or financial relationships that could be construed as a potential conflict of interest.

## Publisher’s note

All claims expressed in this article are solely those of the authors and do not necessarily represent those of their affiliated organizations, or those of the publisher, the editors and the reviewers. Any product that may be evaluated in this article, or claim that may be made by its manufacturer, is not guaranteed or endorsed by the publisher.

## Footnotes

^1^ http://igss.nuaa.edu.cn
